# Effects of vitamin D3, omega-3s, and a simple home exercise program on incident vertebral fractures: the DO-HEALTH randomized controlled trial

**DOI:** 10.1093/jbmr/zjaf058

**Published:** 2025-06-10

**Authors:** Melanie Kistler-Fischbacher, Gabriele Armbrecht, José A P Da Silva, Caroline de Godoi Rezende Costa Molino, Robert Theiler, René Rizzoli, Bruno Vellas, Bess Dawson-Hughes, John A Kanis, Lorenz C Hofbauer, Endel John Orav, Reto W Kressig, Andreas Egli, Guido A Wanner, Heike A Bischoff-Ferrari, DO-HEALTH Research Group, Heike A Bischoff-Ferrari, Andreas Egli, Sandrine Rival, Guido A Wanner, Bruno Vellas, Sophie Guyonnet, René Rizzoli, Emmanuel Biver, Fanny Merminod, W Kressig Reto, Stephanie Bridenbaugh, Norbert Suhm, A P Da Silva José, C M Duarte Cátia, Ana Pinto Filipa, Dieter Felsenberg, Hendrikje Börst, Gabriele Armbrecht, Michael Blauth, Anna Spicher, David T Felson, John A Kanis, Eugene V Mccloskey, Elena Johansson, Bernhard Watzl, Rodriguez Manuel Gomez, Lorenz Hofbauer, Elena Tsourdi, Martina Rauner, Uwe Siebert, John A Kanis, Philippe Halbout, Stephen M Ferrari, Benno Gut, Marième Ba, Jonas Wittwer Schegg, Stéphane Etheve, Manfred Eggersdorfer, Sofa Delannoy Carla, Monika Reuschling, Endel J Orav, C Willett Walter, JoAnn E Manson, Bess Dawson-Hughes, Hannes B Staehelin, W Walter Paul, Walter Dick, Michael Fried, Arnold von Eckardstein, Robert Theiler, Hans-Peter Simmen, Wolfgang Langhans, Annelies Zinkernagel, Nicolas Mueller, Oliver Distler, Klaus Graetz, Ina Nitschke, Thomas Dietrich, Walter Baer, Klara Landau, Frank Ruschitzka, Markus Manz, Peter Burckhardt

**Affiliations:** Department of Aging Medicine and Aging Research, University of Zurich, 8037 Zurich, Switzerland; Centre on Aging and Mobility, University of Zurich and Stadtspital Zurich Waid, 8037 Zurich, Switzerland; Department of Radiology, Charité-Universitätsmedizin Berlin, Corporate Member of Freie Universität Berlin and Humboldt-Universität zu Berlin, Berlin 12203, Germany; Centro Hospitalar e Universitário de Coimbra, Coimbra 3004-561, Portugal; Coimbra Institute for Clinical and Biomedical Research (iCBR), Faculty of Medicine, University of Coimbra, Coimbra 3004-504, Portugal; Department of Aging Medicine and Aging Research, University of Zurich, 8037 Zurich, Switzerland; Centre on Aging and Mobility, University of Zurich and Stadtspital Zurich Waid, 8037 Zurich, Switzerland; Centre on Aging and Mobility, University of Zurich and Stadtspital Zurich Waid, 8037 Zurich, Switzerland; Division of Bone Diseases, Geneva University Hospitals and Faculty of Medicine, 1211 Geneva, Switzerland; Gérontopôle de Toulouse, Institut du Vieillissement, Centre Hospitalo-Universitaire de Toulouse, Toulouse 31059, France; University of Toulouse III, UMR INSERM 1027, Toulouse 31062, France; Jean Mayer USDA Human Nutrition Research Center on Aging, Tufts University, Boston, MA 02111, United States; Center for Metabolic Diseases, University of Sheffield Medical School, Sheffield S10 2TN, United Kingdom; Department of Medicine III, Centre for Healthy Aging, TU Dresden Medical Centre, Dresden 01307, Germany; Department of Biostatistics, Harvard T.H. Chan School of Public Health, Boston, MA 02120, United States; University Department of Geriatric Medicine FELIX PLATTER, 4055 Basel, Switzerland; Department of Aging Medicine and Aging Research, University of Zurich, 8037 Zurich, Switzerland; Centre on Aging and Mobility, University of Zurich and Stadtspital Zurich Waid, 8037 Zurich, Switzerland; Spine Clinic and Traumatology, Private Hospital Bethanien, 8044 Zurich, Switzerland; Department of Aging Medicine and Aging Research, University of Zurich, 8037 Zurich, Switzerland; Centre on Aging and Mobility, University of Zurich and Stadtspital Zurich Waid, 8037 Zurich, Switzerland

**Keywords:** Vertebral morphology, vertebral deformity, vertebral fracture progression, low bone mass, osteoporosis, nutritional supplements, community-dwelling older adults, prevention

## Abstract

Vertebral fractures (VFs) are among the most common osteoporotic fractures. The effect of vitamin D3, omega-3s or a simple home exercise program (SHEP) on VFs is unclear. We examined whether vitamin D3, omega-3s, or SHEP, alone or in combination, over 3 years, reduce the incidence rate of VFs among European older adults. DO-HEALTH is a multi-center, 2 × 2 × 2 factorial design, randomized controlled trial, which included older adults (≥70 years) free from major health events in the 5 years prior to enrollment. The study interventions were vitamin D3 (2000IU/d), omega-3s (1 g/d), and SHEP (3 × 30 min/wk), applied alone or in combination. Quantitative and qualitative VF assessment was determined from lateral thoracolumbar DXA scans. The primary outcome for this analysis was the incidence rate (IR) of total VFs, defined as the number of any new and progressed VFs over the 3-year follow-up. Sensitivity analyses were conducted for only new VFs and only VF progressions. Negative binomial regression models were fit, adjusted for age, sex, prior fall, BMI, study site and participants' follow-up time. 1488 participants (mean age 74.9 years; 77% had low bone mass or osteoporosis; 43.8% had 25(OH)D levels <20 ng/mL) were included. There were 93 incident VFs, of which 58 were new VFs and 35 were progressions. None of the three treatments reduced the IR of total VFs overall, however, the IR was reduced with SHEP compared to the control exercise program in women (IR ratio 0.52, 95% CI 0.28, 0.98). In the sensitivity analysis for VF progressions, SHEP reduced the IR (IR ratio 0.34, 95% CI 0.16, 0.75). Among generally healthy older adults, vitamin D3 and omega-3s supplementation did not reduce the incidence rate of VFs. SHEP reduced the incidence rate of total VFs in women and of VF progressions overall. Exercise may play a role in the prevention of VFs.

## Introduction

Vertebral fractures (VFs) are a hallmark of osteoporosis.^1^^,^^2^ They are highly prevalent^3^ and associated with significant morbidity, including acute and chronic pain, impaired pulmonary function, loss of height and spinal deformities such as thoracic kyphosis.^4–6^ Furthermore, VFs are associated with a 5-fold increased risk of subsequent VFs and a 2-fold increased risk of non-vertebral fracture.^7^

Vitamin D plays a major role in calcium and phosphorus homeostasis, the regulation of parathyroid hormone production and bone turnover and it may thereby support bone health.^8^ While a large number of trials and meta-analyses have examined the effect of vitamin D supplementation on non-vertebral fracture risk,^9–11^ there is little evidence on VF risk. Existing meta-analyses do not support a benefit of vitamin D supplementation for VF risk reduction,^12–15^ but are limited by the relatively small number of included trials (*n* = 4-9), and heterogeneity in terms to co-administration of calcium supplements, dosing regimens (eg, bolus dosing, known to be detrimental for bone health^12^^,^^16^) and VF assessment methods.

Omega-3 fatty acids (omega-3s) affect bone turnover through regulation of vitamin D-dependent intestinal calcium absorption and anti-inflammatory pathways.^17^ The number of randomized controlled trials examining the effect of omega-3s on bone health is limited. Current evidence from RCTs and small meta-analyses does not suggest a benefit of omega-3s supplementation for BMD,^18^ bone turnover markers^19^^,^^20^ or non-vertebral fracture risk.^21^ Data on VFs are lacking.

Exercise can improve bone strength through mechanical loading (mechano-transduction)^22–24^ and reduce the risk of falling,^25^ thereby reducing the risk for fracture. Meta-analyses report a significant risk reduction for major osteoporotic fractures as well as for incident VFs, however, data on the effect of exercise on incident VFs are limited by small sample sizes (eg, <100) and relatively short follow-up duration (6-12 mo).^2^^,^^23^

Based on the distinct mechanisms of action, we hypothesized that the combination of vitamin D3, omega-3s and exercise may exhibit greater benefits for fracture prevention than either alone. However, this hypothesis was rejected for the primary outcome non-vertebral fracture risk in DO-HEALTH, for which none of the interventions showed a benefit.^26^

The present analysis examines the individual and combined effects of vitamin D3 supplementation, omega-3s supplementation and a simple home exercise program on incident VFs, including new VFs and VF progressions, a predefined secondary outcome in DO-HEALTH, assessed in a subsample of participants from four out of seven recruitment sites equipped with DXA machines.

## Materials and methods

### Study design

DO-HEALTH was a 3-year, double-blind, 2 × 2 × 2 factorial design randomized controlled trial. A total of 2157 participants were recruited from seven study centers in five European countries (Switzerland, Germany, Austria, France, and Portugal). The study protocol including the statistical analysis plan has been published.^27^ Incident VFs was a predefined, secondary outcome and assessed in a subsample of participants from four study centers, which were equipped with DXA scanners (Zurich, Switzerland; Berlin, Germany; Toulouse, France; Coimbra, Portugal). Over the 3 years, participants attended yearly clinical visits at baseline, 12, 24, and 36 months. Participants were recruited between December 2012 and November 2014, and the final follow-up visits took place in November 2017.

The trial protocol was registered in the International Trials Registry (clinicaltrials.gov, registration ID: NCT01745263) and approved by regulatory agencies of all countries.^27^

### Study participants

Inclusion criteria were ≥70 years of age, a score of ≥24 on the Mini Mental State Examination, living in the community and sufficient mobility to come to the study centers. Exclusion criteria relevant to osteoporosis and fracture risk included major health events in the 5 years prior to enrolment (eg, cancer, myocardial infarction, and stroke), use of active vitamin D metabolites, PTH treatment (eg, teriparatide) or calcitonin, Paget’s disease of bone, hypo- or hyperparathyroidism, epilepsy or use of anti-convulsive drugs. Furthermore, participants had to be willing to limit calcium supplementation to a maximum dose of 500 mg/d, limit vitamin D supplementation to 800 IU/d and forgo omega-3s supplementation for the duration of the 3-year trial. Having had a fracture within the past 6 weeks was a temporary exclusion criteria and participants were eligible after fracture healing. In order to recruit participants with an increased risk of falling, DO-HEALTH specifically aimed to recruit 40% of participants at each study site who reported a fall in the 12 months prior to enrolment. The full list of eligibility criteria has been published.^27^

### Interventions

This trial had three primary treatment comparisons: vitamin D3 (2000 IU/d) compared to placebo, omega-3s (1 g/d) compared to placebo and a simple home exercise program (SHEP) of 30 min three times per week compared to an attention control exercise program (flexibility exercises) also conducted three times per week.

Vitamin D3 capsules contained 1000 IU of cholecalciferol, stabilized with dl-α-tocopherol (vitamin E, 2.5 permille). Omega-3s capsules contained 500 mg of eicosapentaenoic acid (EPA) and docosahexaenoic acid (DHA) in a ratio of 1:2. Placebo capsules contained sunflower oil and were similar in appearance to vitamin D3 and omega-3s capsules, respectively. For both the vitamin D3 and omega-3s interventions, participants were asked to take 2 capsules a day to achieve the dose of 2000 IU of vitamin D3 and 1 g of omega-3s per day.

The simple home exercise program (SHEP) consisted of the following five exercises: sit-to-stand, single leg stance, pull back against elastic resistance, external shoulder rotation against elastic resistance, and stepping up and down one step on stairs. Three sets of 10 repetitions were performed for each exercise, except for single leg stance, where 10 sets of 10 s were performed for each leg. Participants were allowed to hold on to a stable object (eg, a desk) or the wall during the single leg stance. The attention control was a flexibility exercise program including 5 exercises targeting hip, knee, ankle, trunk, chest, and shoulder. Participants were instructed to perform the exercises 3 times per week.

Adherence to the interventions was assessed by participant self-report at each 3-monthly contact (phone calls and clinical visits) and through capsule counts, which were returned by the participants at each visit.

### Randomization and blinding

Allocation to one of the eight treatment arms was computer-based (DO-HEALTH randomization software), using stratified block (block size of 16 individuals) randomization. Stratification variables were age (70-84 years, ≥85 years), sex, study center, and history of falling in the 12 months prior to enrolment (yes/no). Treatment allocation was concealed and all study staff, data analyses and participants were blinded. The exception was a single physiotherapist who instructed the home exercise programs but was not involved in the trial otherwise.

### Outcome

The primary endpoint of this DO-HEALTH VF analysis was the incidence rate of total osteoporotic VFs, including both new VFs and progression of existing VFs. Sensitivity analyses were conducted for incidence rate of new VFs only (excluding VF progressions) and incidence rate of VF progressions (excluding new VFs).

Lateral thoracolumbar spine scans were performed in the lateral decubitus position, using DXA (Lunar iDXA, GE Healthcare) at each clinical visit (BL, 12, 24, and 36 months). Semi-automated enCORE morphometry analysis software (version 13.60.033) was used for morphometric evaluation of anterior, mid and posterior vertebral height from T4 to L4. Manual correction of measurement points was applied if necessary. A vertebra was considered as deformed, if the height ratio for anterior/posterior or mid/posterior was below 80%. Etiology of vertebral deformities was then classified as osteoporotic (low trauma), traumatic (high trauma), degenerative or other (eg, Morbus Bechterew, hemivertebra) based on published radiological criteria, including reduction of anterior, mid and/or posterior height, height of the disk space, change in vertebral width, presence of spondylophytes, sclerotic endplates, and encroachment of the spinal canal.^28^ For our outcome incident VFs, only deformities that were classified as osteoporotic were included in the analysis. For osteoporotic VFs, the grade of severity as well as shape of fracture was classified using the Genant method^29^: *mild* (grade 1%, 20%-25% reduction in either anterior or middle height relative to posterior height of the same vertebral body, or reduction of posterior height relative to posterior height of adjacent vertebral bodies); *moderate* (grade 2%, 26%-40% reduction in any height); and *severe* (grade 3, >40% reduction in any height). In addition to new fractures, we evaluated the progression of existing fractures. A VF progression was defined as a progression in severity (ie, mild to moderate, mild to severe and moderate to severe) or a progression in deformity type (eg, wedge to crush deformity). For the analyses on VF progression we only included participants with a prevalent VF at baseline, or those with an incident VF at year 1 or year 2.

For participant positioning and scan acquisition standard operating procedures were followed. A designated quality assurance center (Berlin) and experienced radiologist (GA) ensured consistency in scan acquisition among the different densitometry sites and performed all morphometric analyses. The center in Berlin also performed quality control of the DXA machines, as well as training and certification of all staff involved in DO-HEALTH DXA data collection.

### Statistical analyses

Characteristics of study participants are described overall and by treatment group. Normally distributed continuous variables are presented as mean and SD and non-normally distributed variables as median and IQR. Categorical variables are presented as frequencies and percentages. Baseline characteristics were compared between treatment groups using a *t*-test for normally distributed continuous variables, the Wilcoxon test for non-normally distributed continuous variables, and the chi-square test for categorical variables.

The treatment effects on the incidence rate of VFs were analyzed using negative binomial regression models. Three-way and two-way interactions between the treatment groups were tested for each outcome and no significant interactions were found (*p* > .05) for any of the outcomes. Therefore, three dichotomous indicators (ie, the main effect of each treatment) were included in the models. The main effect represents the mean effect of the intervention over the 3-year follow-up.

For the primary outcome, total VFs, predefined subgroup analyses were performed by sex (female, male), age (70-74 vs ≥75 years), and vitamin D deficiency (<20 vs ≥20 ng/mL), median baseline polyunsaturated fatty acid levels (DHA + EPA <100 vs ≥100 μg/mL) and physical activity levels (active <1 vs ≥1/wk). Stratified analysis by subgroups were conducted if a significant interaction (*p* < .05) was found between at least one of the treatment groups and the respective subgroup.

A sensitivity analysis restricted to participants who were not taking bone medications for the outcome total VFs was conducted. In addition, sensitivity analyses were conducted for new VFs only (excluding VF progressions) and VF progressions only (excluding new VFs).

Models were adjusted for sex, age, linear spline at age 85, prior fall, study site, and BMI. Additionally, an offset of the logarithm of each participant’s time in the study was also included to account for exposure time.

Power calculations for the DO-HEALTH trial were based on primary outcomes^27^ and no a priori sample size calculations were conducted for secondary outcomes, including VF. Nevertheless, we conducted *a posterori* power analysis for the outcome total VFs. With a sample size of 1369 participants the study can detect an incidence rate ratio (IRR) of 0.47 with 80% power and a 5% significance level.

The significance level was set at 0.05 (two-sided) and analyses were performed in SAS v9.4 statistical software (Copyright 2004 by SAS Institute Inc.).

## Results

### Study participants

Lateral spine DXA scans were available for 1488 participants at baseline. Of those, 1369 had available DXA scans at follow-up and were included in the analyses. The CONSORT diagram for this subsample of DO-HEALTH participants is shown in Figure S1. The mean age of participants at baseline was 74.9 years, 63.1% were women and 80.7% were physically active at least once per week. The mean comorbidity score was 3.4, the mean femoral neck T-score was −1.4, and 77.0% of participants had low bone mass (osteopenia) or osteoporosis at baseline. Overall, 43.8% of participants were vitamin D deficient (serum 25[OH]D levels <20 ng/mL) and mean 25(OH)D serum levels were 21.9 ng/mL. Baseline characteristics of participants are shown in Table 1. Baseline characteristics by sex are shown in Table S1. Women had a significantly higher comorbidity score, lower T-scores at the lumbar spine and femoral neck, were more likely to have osteopenia or osteoporosis, and had lower physical activity levels compared to men.

**Table 1:** Baseline characteristics of study participants.

		**Vitamin D**			**Omega-3s**			**Exercise**		
**Characteristics**	**Overall (*n* = 1488)**	**Vitamin D (*n* = 742)**	**No vitamin D (*n* = 746)**	***P*** ^***a***^	**Omega-3s (*n* = 745)**	**No omega-3s (*n* = 743)**	***p***	**SHEP (*n* = 746)**	**Control exercise (*n* = 742)**	***p***
**Age [years], mean (SD)** ^**b**^	74.9 (4.4)	75.0 (4.4)	74.9 (4.3)	0.63	74.8 (4.2)	75.0 (4.5)	0.23	75.0 (4.4)	74.8 (4.3)	0.49
**BMI [kg/m** ^**2**^ **], mean (SD)** ^**c**^	26.6 (4.3)	26.7 (4.4)	26.50 (4.2)	0.22	26.6 (4.2)	26.6 (4.3)	0.76	26.6 (4.3)	26.5 (4.3)	0.72
**Women, *n* (%)**	939 (63.1)	473 (63.8)	466 (62.5)	0.61	471 (63.2)	468 (63.0)	0.93	469 (62.9)	470 (63.3)	0.85
**Comorbidity score, mean (SD)** ^**b**^ ^**,**^ ^**d**^	3.4 (3.2)	3.4 (3.2)	3.3 (3.1)	0.57	3.4 (3.3)	3.3 (3.0)	0.63	3.3 (3.2)	3.4 (3.1)	0.37
**Lumbar spine T-score, mean (SD)**	−1.3 (−1.4)	−1.3 (1.4)	−1.3 (1.5)	0.81	−1.4 (1.4)	−1.1 (1.5)	0.001	−1.2 (1.4)	−1.3 (1.5)	0.53
**Femoral neck T-score, mean (SD)**	−1.4 (1.0)	−1.4 (1.0)	−1.4 (1.0)	0.89	−1.5 (1.0)	−1.4 (1.0)	0.04	−1.4 (1.0)	−1.4 (1.0)	0.74
**Bone status based on T-scores** ^**e**^ **, *n* (%)**				0.04			0.21			0.38
**Normal**	336 (23.0)	157 (21.5)	179 (24.4)		162 (22.1)	174 (23.9)		173 (23.6)	163 (22.4)	
**Osteopenia**	792 (54.1)	419 (57.5)	373 (50.8)		390 (53.1)	402 (55.1)		404 (55.0)	388 (53.2)	
**Osteoporosis**	335 (22.9)	153 (21.0)	182 (24.8)		182 (24.8)	153 (21.0)		157 (21.4)	178 (24.4)	
**Bone medication intake, *n* (%)**	186 (12.5)	91 (12.3)	95 (12.7)	0.78	89 (12.0)	97 (13.1)	0.52	88 (11.8)	98 (13.2)	0.41
**Fall history, *n* (%)**	598 (40.2)	297 (40.0)	301 (40.4)	0.90	296 (39.7)	302 (40.7)	0.72	300 (40.2)	298 (40.2)	0.98
**SPPB score, median (IQR)** ^**f**^	11 (10, 12)	11 (10, 12)	11 (10, 12)		11 (10, 12)	11 (10, 12)		11 (10, 12)	11 (10, 12)	0.846
**Physical activity level, *n* (%)**				0.04			0.94			0.82
**Inactive**	287 (19.3)	162 (21.8)	125 (16.8)		141 (19.0)	146 (19.7)		141 (18.9)	146 (19.7)	
**Moderately active (1-3 times/weeks)**	472 (31.7)	225 (30.3)	247 (33.2)		237 (31.9)	235 (31.6)		242 (32.5)	230 (31.0)	
**Active (>3 times/weeks)**	728 (49.0)	355 (47.8)	373 (50.0)		366 (49.2)	362 (48.7)		362 (48.6)	366 (49.3)	
**Serum DHA concentration [μg/mL], mean (SD)**	80.7 (38.3)	80.6 (39.2)	80.8 (37.4)	0.92	81.4 (38.5)	80.1 (38.1)	0.52	80.9 (37.5)	80.6 (39.2)	0.89
**Serum EPA concentration [μg/mL], median (IQR)**	26.9 (18.6, 39.6)	26.2 (18.3, 39.2)	27.3 (19.3, 40.0)	0.16	26.8 (18.7, 39.5)	26.9 (18.4, 40.3)	0.89	26.3 (18.2, 39.4)	27.3 (19.4, 39.9)	0.24
**Serum 25(OH)D concentration [ng/mL], mean (SD)**	21.9 (8.3)	21.9 (8.4)	21.8 (8.3)	0.71	21.8 (8.3)	21.9 (8.4)	0.74	22.3 (8.5)	21.4 (8.2)	0.04
**Vitamin D deficiency (<20 ng/mL), *n* (%)**	646 (43.8)	311 (42.3)	335 (45.2)	0.26	320 (43.4)	326 (44.2)	0.75	312 (42.3)	334 (45.3)	0.25

Abbreviations: BMI, body mass index; DHA, docosahexaenoic acid; EPA, eicosapentaenoic acid; IQR, interquartile range; SD, standard deviation; SHEP, simple home exercise program; SPPB, short physical performance battery.

a *p* values from *t*-test for normally distributed continuous variables, Wilcoxon test for non-normally distributed continuous variables, and the chi-square test for categorical variables.

^b^Median and IQR are presented for non-normally distributed variables.

c BMI was calculated as weight in kilograms divided by height in meters squared.

^d^Comorbidity was measured by the Self-Administered Comorbidity Questionnaire, which assesses 12 comorbidities by three dimensions (presence, medication, and limitation of activities). It has a range of 0-36 points and lower scores indicate better health.

e A T-score at the lumbar spine and/or femoral neck and/or total hip of >1.0 was defined as normal, ≤−1.0 to >−2.5 as osteopenia and ≤−2.5 as osteoporosis.

^f^The SPPB assesses lower extremity function. Scores range from 0 to 12, in which higher scores are better.

Adherence to the study medication and the exercise program for this study sample of 1369 DO-HEALTH participants are presented in Table S2. By year 3, ≥78% participants still took at least 80% of their total study medication and ≥65% performed the SHEP at least twice per week.

### Outcome total osteoporotic VFs

Over the 3-year follow-up among 1369 participants, there were a total of 93 VFs, of which 58 were new VFs and 35 were progressions of existing VFs. The 58 new VFs occurred in 50 participants, of which 42 had a single incident VF and 8 had 2 incident VFs. The 35 VF progressions occurred in 31 out of 157 participants with prevalent fractures. Of the 31 participants, 28 sustained one incident VF progression, 2 participants sustained 2 VF progressions, and 1 participant sustained 3 VF progressions.

**Figure 1:**
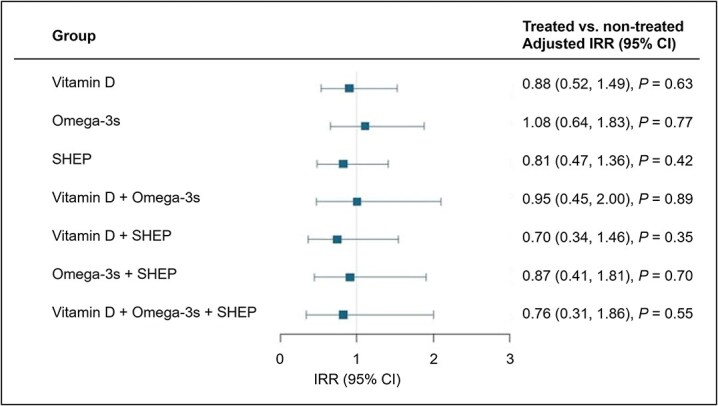
Treatment effects on the incidence rate of total VFs over the 3-year follow-up. Analyses adjusted for age, linear spline at age 85 years, sex, prior fall, BMI, and study site. *N* = 1369*.*

For the outcome total VFs, there were no 3- or 2-way treatment interactions. Therefore, results from main effect analyses are presented and treatment effects are additive. For vitamin D3, omega-3s and SHEP, there were no significant differences in total VF between treatment and comparison groups across the 3-year follow (Table 2). Similarly, there were no significant effects for treatment combinations (Figure 1). There was a significant interaction for the subgroup by sex and SHEP (*p* = .04, Table S3) suggesting a possible benefit among women (IRR = 0.52, 95% CI 0.28, 0.98) and no benefit among men (IRR = 2.27, 95% CI 0.81, 6.38; Table S4). Furthermore, there was a significant interaction for the subgroup by vitamin D deficiency and omega-3s (*p* = .04, Table S3) but there were no significant treatment effects for omega-3s in those with vitamin D deficiency (IRR = 0.69, 95% CI 0.29, 1.63) or those with vitamin D sufficiency (IRR = 1.63, 95% CI 0.82, 3.22; Table S5). There were no significant interactions between baseline physical activity or serum PUFA levels and any of the 3 treatments (Table S3).

**Table 2:** Treatment effects on the incidence rate of total VFs (new and progressed).

	**Vitamin D3**	**No vitamin D3**	**Omega-3s**	**No omega-3s**	**SHEP**	**Control exercise**
**No. of participants**	680	689	683	686	681	688
**No. of total VFs**	44	49	46	47	41	52
**Incidence rate per person-year**^**a**^	0.02 (0.02, 0.03)	0.02 (0.02, 0.03)	0.02 (0.02, 0.03)	0.02 (0.02, 0.03)	0.02 (0.01, 0.03)	0.03 (0.02, 0.04)
**Incidence rate ratio**^**a**^	0.91 (0.54, 1.54)		0.99 (0.58, 1.67)		0.79 (0.46, 1.33)	
***p* value**^**a**^	0.73		0.96		0.37	
**Adjusted estimates** ^**b**^						
**Adjusted incidence rate per person-year (95% CI)**	0.02 (0.01, 0.03)	0.02 (0.01, 0.03)	0.02 (0.01, 0.03)	0.02 (0.01, 0.03)	0.02 (0.01, 0.02)	0.02 (0.01, 0.03)
**Adjusted incidence rate ratio (95% CI)**	0.88 (0.52, 1.49)		1.08 (0.64, 1.83)		0.81 (0.47, 1.36)	
***p* value**	0.63		0.77		0.42	

Abbreviations: CI, confidence interval; SHEP, simple home exercise program; VF, vertebral fracture.

^a^Rates and *p* values are from negative binomial regression models, adjusted for participants' follow-up times, but not for covariates.

^b^Rates and *p* values from negative binominal regression models, adjusted for participants' follow-up times, age, linear spline at age 85 years, sex, prior fall, BMI, study site.

### Results from sensitivity analyses

For all sensitivity analyses there were no 3- or 2-way treatment interactions. For sensitivity analyses, only main effects are shown, without treatment combinations.

For new VFs only, there were no significant differences between treatment and comparison groups across the 3-year follow up (Table 3).

**Table 3:** Treatment effects on the incidence rate of new VFs, excluding VF progressions, results from sensitivity analyses.

	**Vitamin D3**	**No vitamin D3**	**Omega-3s**	**No omega-3s**	**SHEP**	**Control exercise**
**No. of participants**	680	689	683	686	681	688
**No. of new VFs**	28	30	30	28	31	27
**Incidence rate per person-year**^**a**^	0.01 (0.01, 0.02)	0.01 (0.01, 0.02)	0.01 (0.01, 0.02)	0.01 (0.01, 0.02)	0.02 (0.01, 0.02)	0.01 (0.01, 0.02)
**Incidence rate ratio**^**a**^	0.95 (0.53, 1.70)		1.08 (0.60, 1.94)		1.15 (0.64, 2.06)	
***p* value**^**a**^	0.85		0.80		0.65	
**Adjusted estimates** ^**b**^						
**Adjusted incidence rate per person-year (95% CI)**	0.01 (0.01, 0.02)	0.01 (0.01, 0.02)	0.01 (0.01, 0.02)	0.01 (0.01, 0.02)	0.01 (0.01, 0.02)	0.01 (0.01, 0.02)
**Adjusted incidence rate ratio (95% CI)**	0.93 (0.52, 1.67)		1.13 (0.63, 2.02)		1.09 (0.61, 1.96)	
***p* value**	0.81		0.68		0.77	

Abbreviations: CI, confidence interval; SHEP, simple home exercise program; VF, vertebral fracture.

^a^Rates and *p* values are from negative binomial regression models, adjusted for participants' follow-up times, but not for covariates.

^b^Rates and *p* values from negative binominal regression models, adjusted for participants' follow-up times, age, linear spline at age 85 years, sex, prior fall, BMI, study site.

For VF progressions only, there were no significant effects for Vitamin D and omega-3s, whereas SHEP significantly reduced the incidence rate of VF progressions (IRR = 0.34, 95% CI 0.16, 0.75; Table 4) compared to the control exercise program.

**Table 4:** Treatment effects on the incidence rate of VF progressions, excluding new VFs, results from sensitivity analyses.

	**Vitamin D3**	**No vitamin D3**	**Omega-3s**	**No omega-3s**	**SHEP**	**Control exercise**
**No. of participants**	79	78	83	74	83	74
**No. of VF progressions**	16	19	16	19	10	25
**Incidence rate per person-year**^**a**^	0.08 (0.05, 0.12)	0.09 (0.06, 0.15)	0.08 (0.05, 0.12)	0.09 (0.06, 0.15)	0.04 (0.02, 0.08)	0.13 (0.09, 0.19)
**Incidence rate ratio**^**a**^	0.81 (0.41, 1.61)		0.82 (0.41, 1.62)		0.34 (0.16, 0.72)	
***p* value**^**a**^	0.55		0.56		0.005	
**Adjusted estimates** ^**b**^						
**Adjusted incidence rate per person-year (95% CI)**	0.06 (0.03, 0.11)	0.07 (0.04, 0.11)	0.06 (0.03, 0.10)	0.07 (0.04, 0.12)	0.04 (0.02, 0.07)	0.11 (0.07, 0.18)
**Adjusted incidence rate ratio (95% CI)**	0.92 (0.46, 1.84)		0.78 (0.40, 1.54)		0.34 (0.16, 0.75)	
***p* value**	0.81		0.47		0.007	

Abbreviations: CI, confidence interval; SHEP, simple home exercise program; VF, vertebral fracture.

a Rates and *p* values are from negative binomial regression models, adjusted for participants' follow-up times, but not for covariates.

b Rates and *p* values from negative binominal regression models, adjusted for participants' follow-up times, age, linear spline at age 85 years, sex, prior fall, BMI, study site.

For the sensitivity analysis on total VFs, excluding participants who were taking bone medications at baseline, there were no significant differences between treatment and comparison groups across the 3-year follow up (Table S6).

## Discussion

The purpose of the present study was to examine the independent and combined effects of daily vitamin D3 supplementation, daily omega-3s supplementation and/or a thrice-weekly exercise program on the incidence rate of VFs over a 3-year follow-up period in a subsample of 1369 DO-HEALTH participants. Supplementation with 2000 IU vitamin D3 or 1 g of omega-3s per day showed no benefit on the incidence rate of total, new or progression of VFs. The simple home exercise program had no effect on the incidence rate of total or new VFs, however, it significantly reduced the incidence rate of VF progressions.

Several meta-analyses report beneficial effects of exercise, and particularly strength and resistance training, on reduction of major osteoporotic fractures or non-vertebral fractures.^2^^,^^23^^,^^30^ However, few trials have examined and reported exercise effects on VFs. A small meta-analysis of three trials concluded a 44% reduction in VFs among older adults, however, results did not reach statistical significance (RR = 0.56, 95% CI 0.30-1.04).^2^ Our findings confirm a positive effect of exercise on total VFs in women. Women had lower lumbar spine and femoral neck T-scores at baseline, were more likely to have osteopenia or osteoporosis, were less physically active and had a higher comorbidity score than men. These factors may have contributed to the positive effects detected in women but not men. Furthermore, we observed a positive effect of SHEP in the analyses restricted to VF progression. Little is known about the progression patterns and clinical risk factors for progression of VFs,^31^ and to the best of our knowledge, this is the first trial to examine exercise effects on VF progression. Our results thus add new insights to existing evidence on the potential role of exercise for the prevention of osteoporotic VFs.^32^^,^^33^ It is possible that overall, the intensity of the SHEP tested in DO-HEALTH was insufficient to stimulate an effect on BMD^18^ and to reduce falls^34^ and non-vertebral fracture risk^26^ among healthy and active older adults, but was sufficient to prevent VF progression. While we can only speculate about the mechanism by which SHEP reduced VF progression, it is possible that SHEP may have exerted positive effects on back extensor strength and spine mobility (2 factors that were not assessed in DO-HEALTH) and thus prevented VF progression. Overall, and although our results were only significant in sensitivity and subgroup analysis, they align with the well acknowledged benefits of exercise for fracture prevention.^23^^,^^30^

In this trial, supplemental vitamin D3 did not reduce the rate of VFs. This finding aligns with the results for the DO-HEALTH primary outcome non-vertebral fractures^26^ and the findings from the Vitamin D and Omega-3 Trial (VITAL).^35^ The VITAL trial tested the same dose of 2000 IU vitamin D3 over a median follow-up of 5.3 years, included 25 871 participants (mean age 67 years) and reported no benefits for total, non-vertebral and hip fracture risk.^11^ Importantly, DO-HEALTH and VITAL were primary prevention trials and included middle-aged and older adults, unselected for vitamin D deficiency, osteoporosis or fracture risk. The evidence from these 2 large-scale trials and recent meta-analyses^14^^,^^36^^,^^37^ do not support the use of vitamin D supplements for primary prevention of osteoporotic fractures in vitamin D replete adults. However, vitamin D deficiency is associated with reduced bone strength and an increased fracture risk.^36^^,^^38^ Although in the present trial, the subgroup analyses by vitamin D deficiency did not yield any significant effects for vitamin D supplementation, previous studies have shown that daily supplementation with 600-800 IU vitamin D can correct vitamin D deficiency and significantly reduce fracture risk in individuals with vitamin D deficiency or high fracture risk.^39–41^ Our findings thus do not challenge the current recommended daily intake of 800-1000 IU for those older adults with high fracture risk.^40^^,^^42^ Further, our prior data do support a benefit of 2000 IU vitamin D in DO-HEALTH regarding hip BMD.^18^

The mechanistic link between omega-3s and bone metabolism as well as epidemiological data have led to interest in the effect of omega-3s supplementation on fracture risk. For example, observational data from the Women’s Health initiative suggests a 54% lower risk for hip fracture among those with the highest compared to the lowest EPA levels.^43^ Until recently, data from large clinical trials testing the effect of omega-3s supplementation on bone health and fracture risk were lacking. However, both the DO-HEALTH (*N* = 2157) and the VITAL (*N* = 25 871) trials showed no benefit of daily omega-3s supplementation on non-vertebral fracture risk,^21^^,^^26^ BMD (assessed in a subsample in both trials)^18^^,^^35^ or vertebral fracture risk, as shown in the present study. Furthermore, there were no benefits of omega-3s on bone turnover markers, including sclerostin, P1NP or CTx in DO-HEALTH.^44^ Current evidence therefore does not support omega-3s supplementation to improve bone health in generally healthy middle-aged to older adults.

This study has some important strengths. First, the sample size and follow-up duration exceed that of many previous trials, particularly those on omega-3s supplementation and exercise. Second, the methodology for VF assessment follows the current guidelines of the International Society on Clinical Densitometry and included both morphology as well as visual evaluation. Furthermore, all DXA images across the four study centres were assessed by a single and highly experienced radiographer, which ensures reliability of the assessments.

We also acknowledge some limitations of the present study. First, participants were physically active at baseline, were not selected for low bone mass or increased fracture risk, nor where they selected for vitamin D deficiency. This reflects a conservative approach and may have limited the ability to detect significant treatment effects. Second, in line with current guidelines, all participants were allowed to take up to 800 IU/d of supplemental vitamin D3, outside the trial interventions. Third, the sample size for VF progressions was small. Furthermore, generalization of findings to populations at higher fracture risk is limited. And last, *p* values were not adjusted for multiple testing and consideration of multiple comparisons is warranted when interpreting our findings.

In conclusion, vitamin D3 and omega-3s supplements did not reduce the incidence rate of total VFs and new VFs over the 3-year follow-up among generally healthy and active older adults. The simple home exercise program reduced the incidence rate of total VFs in women and of VF progressions overall. Exercise may play a role in the prevention of VFs, however, further trials are needed to confirm these findings in a larger sample, particularly for VF progressions.

### DO-HEALTH Research Group

The members of DO-HEALTH Research Group are Bischoff-Ferrari Heike A., Egli Andreas, Rival Sandrine, Wanner Guido A., Vellas Bruno, Guyonnet Sophie, Rizzoli René, Biver Emmanuel, Merminod Fanny, Kressig Reto W., Bridenbaugh Stephanie, Suhm Norbert, Da Silva José A.P., Duarte Cátia C.M., Pinto Filipa Ana, Felsenberg Dieter, Börst Hendrikje, Armbrecht Gabriele, Blauth Michael, Spicher Anna, Felson David T., Kanis John A., Mccloskey Eugene V., Johansson Elena, Watzl Bernhard, Gomez Rodriguez Manuel, Hofbauer Lorenz, Tsourdi Elena, Rauner Martina, Siebert Uwe, Kanis John A., Halbout Philippe, Ferrari Stephen M., Gut Benno, Ba Marième, Wittwer Schegg Jonas, Etheve Stéphane, Eggersdorfer Manfred, Delannoy Carla Sofa, Reuschling Monika, Orav Endel J., Willett Walter C., Manson JoAnn E., Dawson-Hughes Bess, Staehelin Hannes B., Walter Paul W., Dick Walter, Fried Michael, von Eckardstein Arnold, Theiler Robert, Simmen Hans-Peter, Langhans Wolfgang, Zinkernagel Annelies, Mueller Nicolas, Distler Oliver, Graetz Klaus, Nitschke Ina, Dietrich Thomas, Baer Walter, Landau Klara, Ruschitzka Frank, Manz Markus, and Burckhardt Peter.

## Supplementary Material

Supplementary_Material_DoH_VF_May2025_zjaf058

## Data Availability

The datasets generated and/or analyzed during the current study are available from the corresponding author on reasonable request.
