# Supplementary material for: Effects of vitamin D3, omega-3s, and a simple home exercise program on incident vertebral fractures: the DO-HEALTH randomized controlled trial
Source: J Bone Miner Res. 2025 Jun 10;40(9):1035–44. doi: 10.1093/jbmr/zjaf058 (PMC12406121; doi:10.1093/jbmr/zjaf058)
Supplement: Supplementary_Material_DoH_VF_May2025_zjaf058 [file supplementary_material_doh_vf_may2025_zjaf058.docx]

# SUPPLEMENTARY MATERIAL

**Effects of vitamin D3, omega-3s and a simple home exercise program on incident vertebral fractures: the DO-HEALTH randomized controlled trial**

Melanie Kistler-Fischbacher^1,2*^, Gabriele Armbrecht^3*^, José A.P. Da Silva^4^, Caroline De Godoi Rezende Costa Molino^1,2^, Robert Theiler^2^, René Rizzoli^5^, Bruno Vellas^6^, Bess Dawson-Hughes^7^, John A. Kanis^8^, Lorenz C. Hofbauer^9^, E. John Orav^10^, Reto W. Kressig^11^, Andreas Egli^1,2^, Guido A. Wanner^12^, Heike A. Bischoff-Ferrari^1,2^; DO-HEALTH Research Group

*equal contributions

^1^ Department of Aging Medicine and Aging Research, University of Zurich, Zurich, Switzerland

^2^ Centre on Aging and Mobility, University of Zurich and Stadtspital Zurich Waid, Zurich, Switzerland

^3^ Department of Radiology, Charité-Universitätsmedizin Berlin, Corporate Member of Freie Universität Berlin and Humboldt-Universität zu Berlin, Berlin, Germany

^4^ Centro Hospitalar e Universitário de Coimbra, Coimbra, Portugal; Coimbra Institute for Clinical and Biomedical Research (iCBR), Faculty of Medicine, University of Coimbra, Coimbra, Portugal

^5^ Division of Bone Diseases, Geneva University Hospitals and Faculty of Medicine, Geneva, Switzerland

^6^ Gérontopôle de Toulouse, Institut du Vieillissement, Centre Hospitalo-Universitaire de Toulouse, Toulouse, France; UMR INSERM 1027, University of Toulouse III, Toulouse, France.

^7^ Jean Mayer USDA Human Nutrition Research Center on Aging, Tufts University, Boston, Massachusetts

^8^ Center for Metabolic Diseases, University of Sheffield Medical School, Sheffield, England

^9^ Centre for Healthy Aging, Department of Medicine III, TU Dresden Medical Centre, Dresden, Germany

^10^ Department of Biostatistics, Harvard T.H. Chan School of Public Health, Boston, MA, USA

^11^ University Department of Geriatric Medicine FELIX PLATTER, Basel, Switzerland

^12^ Spine Clinic and Traumatology, Private Hospital Bethanien, Zurich, Switzerland

**SUPPLEMENTAL TABLES**

## Supplemental Table 1. Characteristics of study participants at baseline, by sex

| Characteristics | Overall  (n = 1488) | Women  (n = 939) | Men  (n = 579) | *P ^a^* |
| --- | --- | --- | --- | --- |
| Age [yrs], mean (SD) ^b^ | 74.9 (4.4) | 74.7 (4.4) | 75.2 (4.3) | 0.06 |
| BMI [kg/m^2^], mean (SD) ^c^ | 26.6 (4.3) | 26.5 (4.7) | 26.8 (3.5) | 0.22 |
| Comorbidity score, mean (SD) ^b,d^ | 3.4 (3.2) | 3.7 (3.3) | 2.8 (2.8) | < 0.001 |
| Lumbar spine T-score, mean (SD) | -1.3 (-1.4) | -1.7 (1.3) | -0.6 (1.4) | < 0.001 |
| Femoral neck T-score, mean (SD) | -1.4 (1.0) | -1.5 (1.0) | -1.3 (1.0) | 0.002 |
| Bone status based on femoral neck T-score ^e^ |  |  |  | < 0.001 |
| Normal, n (%) | 336 (23.0) | 162 (17.6) | 174 (32.2) |  |
| Osteopenia, n (%) | 792 (54.1) | 507 (54.9) | 285 (52.8) |  |
| Osteoporosis, n (%) | 335 (22.9) | 254 (27.5) | 81 (15.0) |  |
| Bone medication intake, n (%) | 186 (12.5) | 182 (19.4) | 4 (0.7) | < 0.001 |
| Fall history, n (%) | 598 (40.2) | 420 (44.7) | 178 (32.4) | < 0.001 |
| SPPB score, median (IQR) ^f^ | 11 (10, 12) | 11 (10, 12) | 11 (10, 12) | 0.007 |
| Physical activity level, n (%) |  |  |  | 0.02 |
| Inactive | 287 (19.3) | 193 (20.6) | 94 (17.2) |  |
| Moderately  active (1-3  times/week) | 472 (31.7) | 313 (33.3) | 159 (29.0) |  |
| Active (>3  times/week) | 728 (49.0) | 433 (46.1) | 295 (53.8) |  |
| Serum DHA concentration [µg/mL], mean (SD) | 80.7 (38.3) | 82.3 (39.1) | 78.1 (36.8) | 0.04 |
| Serum EPA concentration [µg/mL], median (IQR) | 26.9  (18.6, 39.6) | 27.8 (19.1, 40.0) | 25.1 (18.2, 38.8) | 0.06 |
| Serum 25-hydroxyvitamin D concentration [ng/mL], mean (SD) | 21.9 (8.3) | 22.2 (8.4) | 21.3 (8.1) | 0.04 |
| Vitamin D deficiency (< 20 ng/mL), n (%) | 646 (43.8) | 381 (41.1) | 265 (48.4) | 0.006 |
| Abbreviations: BMI, body mass index; DHA, docosahexaenoic acid; EPA, eicosapentaenoic acid IQR, interquartile range; SHEP, simple home exercise program; SPPB, short physical performance battery; yrs, years  ^a^ P values from T test for normally distributed continuous variables, Wilcoxon test for non-normally distributed continuous variables, and the chi-square test for categorical variables  ^b^ Median and IQR are presented for non-normally distributed variables  ^c^ Body mass index (BMI) was calculated as weight in kilograms divided by height in meters squared  ^d^ Comorbidity was measured by the Self-Administered Comorbidity Questionnaire, which assesses 12 comorbidities by 3 dimensions (presence, medication, and limitation of activities). It has a range of 0 to 36 points and lower scores indicate better health  ^e^ A T-score at the lumbar spine and/or femoral neck and/or total hip of >1.0 was defined as normal, ≤ -1.0 to > -2.5 as osteopenia and ≤ -2.5 as osteoporosis  ^f^ The Short Physical Performance Battery (SPPB) assesses lower extremity function. Scores range from 0 to 12, in which higher scores are better. | | | | |

## Supplemental Table 2. Adherence to study medication and exercise programs (n, %) by treatment groups

|  | **Took at least 80% of pills** | | | | **Performed exercise ≥2/wk** | | **Performed exercise  ≥ 3/wk** | |
| --- | --- | --- | --- | --- | --- | --- | --- | --- |
| **Time** | **Vitamin D** | **No**  **Vitamin D** | **Omega-3s** | **No**  **Omega-3s** | **SHEP** | **Control exercise** | **SHEP** | **Control exercise** |
| Year 1 | 637/678  (94.0%) | 619/686  (90.2%) | 630/680  (92.7%) | 626/684  (91.5%) | 535/679  (78.8%) | 562/685  (82.0%) | 483/679  (71.1%) | 518/685  (75.6%) |
| Year 2 | 593/666  (89.0%) | 576/668  (86.2%) | 589/666  (88.4%) | 580/668  (86.8%) | 494/664  (74.4%) | 484/670  (72.2%) | 439/664  (66.1%) | 445/670  (66.4%) |
| Year 3 | 560/660  (84.9%) | 530/663  (79.9%) | 556/659  (84.4%) | 534/664  (80.4%) | 440/662  (66.5%) | 450/661  (68.1%) | 389/681  (58.8%) | 415/661 (62.8%) |
| Abbreviations: SHEP, simple home exercise program; wk, week  Includes participants who answered the compliance questionnaire at least once during the study period | | | | | | | | |

## Supplemental Table 3. P values for subgroup interactions for total VFs

|  | **Vitamin D3** | **Omega-3s** | **SHEP** |
| --- | --- | --- | --- |
| Sex (men, women) | 0.69 | 0.67 | 0.04 |
| Age (70-74 vs. ≥75 years) | 0.60 | 0.84 | 0.39 |
| Vitamin D deficiency (< 20 vs. ≥ 20 ng/mL) | 0.54 | 0.04 | 0.71 |
| DHA plus EPA (<100 vs. ≥100 μg/mL) | 0.20 | 0.72 | 0.83 |
| Physical activity (active <1/wk vs. ≥ 1/wk) | 0.74 | 0.58 | 0.94 |
| Abbreviations: DHA, docosahexaenoic acid; EPA, eicosapentaenoic acid; SHEP, simple home exercise program  P values from negative binominal regression models, including interaction term for respective subgroup and the outcome, adjusted for participants’ follow-up times, age, linear spline at age 85 years, sex, prior fall, BMI, study site | | | |

## Supplemental Table 4. Results for the number of total VFs, by subgroup of sex

|  | **Vitamin D3** | **No vitamin D3** | **Omega-3s** | **No omega-3s** | **SHEP** | **Control exercise** |
| --- | --- | --- | --- | --- | --- | --- |
| **Women** |  |  |  |  |  |  |
| No. of participants | 433 | 428 | 429 | 432 | 426 | 435 |
| No. of total VFs | 33 | 37 | 35 | 35 | 25 | 45 |
| Incidence rate per person-year ^a^ | 0.03 (0.02, 0.04) | 0.03 (0.02, 0.05) | 0.03 (0.02, 0.04) | 0.03 (0.02, 0.04) | 0.02 (0.01, 0.03) | 0.04 (0.02, 0.05) |
| Incidence rate ratio ^a^ | 0.89 (0.47, 1.66) |  | 1.02 (0.55, 1.91) |  | 0.56 (0.30, 1.06) |  |
| *P* value ^a^ | 0.70 |  | 0.95 |  | 0.07 |  |
| Adjusted estimates ^b^ |  |  |  |  |  |  |
| Adjusted incidence rate per  person-y (95% CI) | 0.02 (0.01, 0.03) | 0.02 (0.01, 0.04) | 0.02 (0.01, 0.04) | 0.02 (0.01, 0.03) | 0.02 (0.01, 0.03) | 0.03 (0.02, 0.05) |
| Adjusted incidence rate ratio  (95% CI) | 0.86 (0.46, 1.61) |  | 1.16 (0.62, 2.16) |  | 0.52 (0.28, 0.98) |  |
| *P* value | 0.64 |  | 0.64 |  | 0.04 |  |
| **Men** |  |  |  |  |  |  |
| No. of participants | 247 | 261 | 254 | 254 | 255 | 253 |
| No. of total VFs | 11 | 12 | 11 | 12 | 16 | 7 |
| Incidence rate per person-year ^a^ | 0.02 (0.01, 0.03) | 0.02 (0.01, 0.03) | 0.01 (0.01, 0.03) | 0.02 (0.01, 0.03) | 0.02 (0.01 0.04) | 0.01 (0.00, 0.02) |
| Incidence rate ratio ^a^ | 0.97 (0.37, 2.53) |  | 0.90 (0.34, 2.36) |  | 2.23 (0.82, 6.12) |  |
| *P* value ^a^ | 0.94 |  | 0.83 |  | 0.12 |  |
| Adjusted estimates ^b^ |  |  |  |  |  |  |
| Adjusted incidence rate per  person-y (95% CI) | 0.01 (0.01, 0.03) | 0.02 (0.01, 0.03) | 0.01 (0.01, 0.03) | 0.01 (0.01, 0.03) | 0.02 (0.01, 0.04) | 0.01 (0.00, 0.02) |
| Adjusted incidence rate ratio  (95% CI) | 0.85 (0.31, 2.27) |  | 0.93 (0.35, 2.44) |  | 2.27 (0.81, 6.38) |  |
| *P* value | 0.74 |  | 0.88 |  | 0.12 |  |
| Abbreviations: CI, confidence interval; SHEP, simple home exercise program; VFs, vertebral fractures  ^a^ Rates (95% CIs) are from negative binomial regression models, adjusted for participants’ follow-up times, but not for covariates  ^b^ Rates and *P* values from negative binominal regression models, adjusted for participants’ follow-up times, age and BMI | | | | | | |

## Supplemental Table 5. Results for the number of total VFs, by subgroup of vitamin D deficiency

|  | **Vitamin D3** | **No vitamin D3** | **Omega-3s** | **No omega-3s** | **SHEP** | **Control exercise** |
| --- | --- | --- | --- | --- | --- | --- |
| **25(OH)D < 20 ng/mL** |  |  |  |  |  |  |
| No. of participants | 280 | 306 | 292 | 294 | 278 | 308 |
| No. of total VFs | 22 | 22 | 17 | 27 | 20 | 24 |
| Incidence rate per person-year ^a^ | 0.03 (0.01, 0.05) | 0.02 (0.01, 0.04) | 0.02 (0.01, 0.04) | 0.03 (0.02, 0.05) | 0.02 (0.01, 0.45) | 0.03 (0.01, 0.05) |
| Incidence rate ratio ^a^ | 1.08 (0.46, 2.51) |  | 0.63 (0.27, 1.47) |  | 0.91 (0.39, 2.13) |  |
| *P* value ^a^ | 0.86 |  | 0.28 |  | 0.84 |  |
| Adjusted estimates ^b^ |  |  |  |  |  |  |
| Adjusted incidence rate per  person-y (95% CI) | 0.02 (0.01, 0.04) | 0.02 (0.01, 0.03) | 0.01 (0.01, 0.03) | 0.02 (0.01, 0.04) | 0.02 (0.01, 0.03) | 0.02 (0.01, 0.04) |
| Adjusted incidence rate ratio  (95% CI) | 1.00 (0.42, 2.41) |  | 0.69 (0.29, 1.63) |  | 0.85 (0.35, 2.09) |  |
| *P* value | 0.99 |  | 0.40 |  | 0.73 |  |
| **25(OH)D ≥ 20 ng/mL** |  |  |  |  |  |  |
| No. of participants | 395 | 378 | 386 | 387 | 397 | 376 |
| No. of total VFs | 22 | 27 | 29 | 20 | 21 | 28 |
| Incidence rate per person-year ^a^ | 0.02 (0.01, 0.03) | 0.02 (0.02, 0.04) | 0.03 (0.02, 0.04) | 0.02 (0.01, 0.03) | 0.02 (0.01, 0.03) | 0.03 (0.02, 0.04) |
| Incidence rate ratio ^a^ | 0.79 (0.40, 1.53) |  | 1.47 (0.75, 2.88) |  | 0.70 (0.36, 1.37) |  |
| *P* value ^a^ | 0.48 |  | 0.26 |  | 0.30 |  |
| Adjusted estimates ^b^ |  |  |  |  |  |  |
| Adjusted incidence rate per  person-y (95% CI) | 0.02 (0.01, 0.03) | 0.02 (0.01, 0.04) | 0.02 (0.01, 0.04) | 0.01 (0.01, 0.03) | 0.02 (0.01, 0.03) | 0.02 (0.01, 0.04) |
| Adjusted incidence rate ratio  (95% CI) | 0.75 (0.38, 1.47) |  | 1.63 (0.82, 3.22) |  | 0.72 (0.37, 1.43) |  |
| *P* value | 0.40 |  | 0.16 |  | 0.35 |  |
| Abbreviations: CI, confidence interval; SHEP, simple home exercise program; VFs, vertebral fractures  ^a^ Rates (95% CIs) are from negative binomial regression models, adjusted for participants’ follow-up times, but not for covariates  ^b^ Rates and *P* values from negative binominal regression models, adjusted for participants’ follow-up times, age, sex, BMI, prior fall, and study site. | | | | | | |

## Supplemental Table 6. Treatment effects on the incidence rate of total VFs, excluding participants taking osteoporosis medications ^a^, results from sensitivity analyses

|  | **Vitamin D3** | **No vitamin D3** | **Omega-3s** | **No omega-3s** | **SHEP** | **Control exercise** |
| --- | --- | --- | --- | --- | --- | --- |
| No. of participants | 600 | 603 | 605 | 598 | 606 | 597 |
| No. of total VFs | 39 | 44 | 40 | 43 | 37 | 46 |
| Incidence rate per person-year ^b^ | 0.02 (0.01, 0.03) | 0.02 (0.02, 0.04) | 0.02 (0.02, 0.03) | 0.02 (0.02, 0.04) | 0.02 (0.01, 0.03) | 0.03 (0.02, 0.04) |
| Incidence rate ratio ^b^ | 0.89 (0.51, 1.57) |  | 0.92 (0.53, 1.62) |  | 0.78 (0.45, 1.37) |  |
| *P* value ^b^ | 0.70 |  | 0.78 |  | 0.39 |  |
| Adjusted estimates ^c^ |  |  |  |  |  |  |
| Adjusted incidence rate per  person-y (95% CI) | 0.02 (0.01, 0.03) | 0.02 (0.01, 0.03) | 0.02 (0.01, 0.03) | 0.02 (0.01, 0.03) | 0.02 (0.01, 0.02) | 0.02 (0.01, 0.03) |
| Adjusted incidence rate ratio  (95% CI) | 0.84 (0.48, 1.48) |  | 1.01 (0.58, 1.76) |  | 0.80 (0.46, 1.41) |  |
| *P* value | 0.55 |  | 0.98 |  | 0.44 |  |
| Abbreviations: CI, confidence interval; SHEP, simple home exercise program; VFs, vertebral fractures  ^a^ Osteoporosis medications were defined as bisphosphonates, denosumab, strontium ranelate, selective estrogen receptor modulators, hormone therapy (estrogen or combined estrogen plus progesterone therapy)  ^b^ Rates and *P* values are from negative binomial regression models, adjusted for participants’ follow-up times, but not for covariates  c Rates and *P* values from negative binominal regression models, adjusted for participants’ follow-up times, age, linear spline at age 85 years, sex, prior fall, BMI, study site | | | | | | |

## SUPPLEMENTAL FIGURES


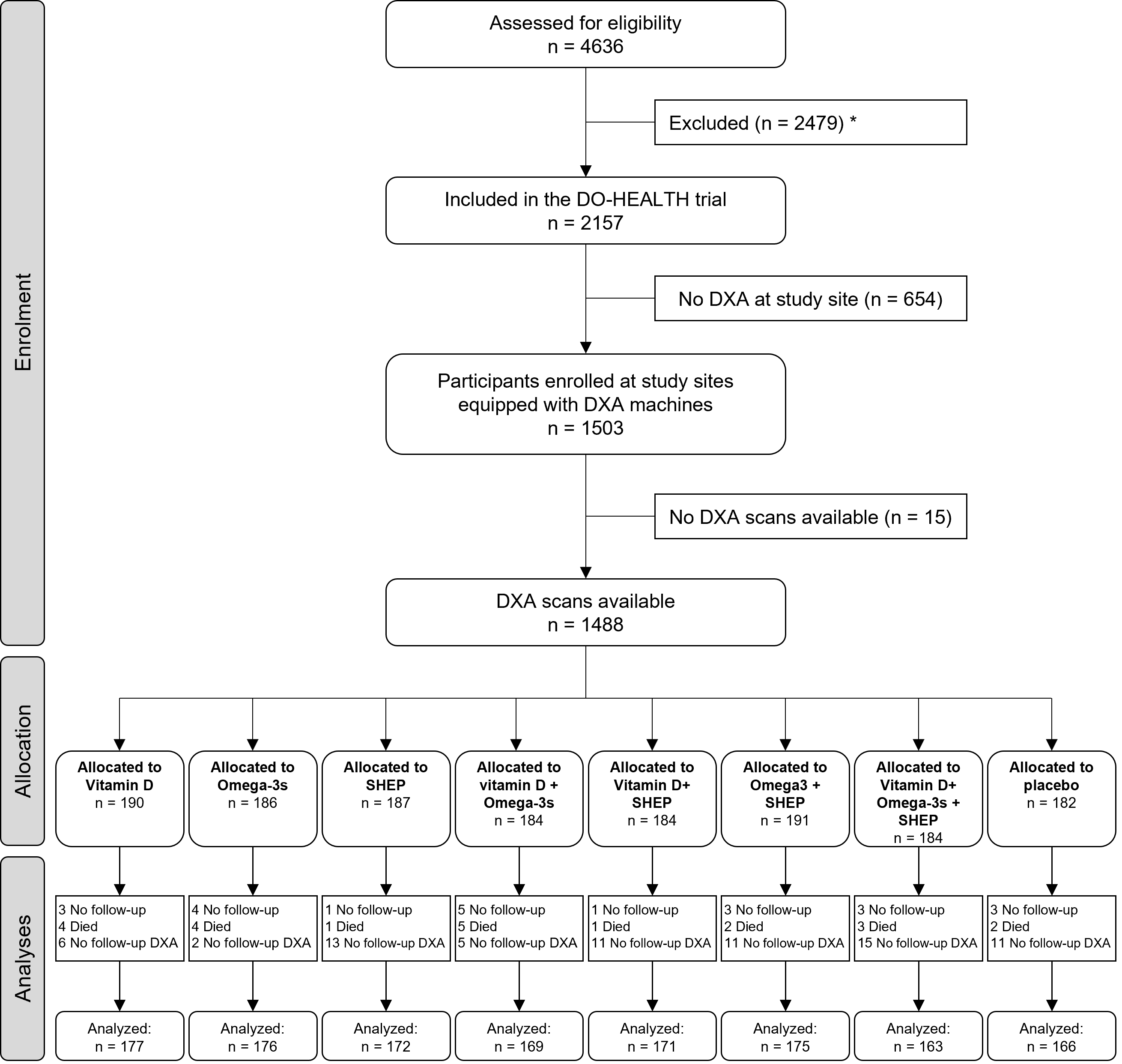


## Supplemental Figure 1. CONSORT flow diagram of DO-HEALTH participants undergoing lateral thoracolumbar spine DXA scans. *Reasons for exclusion are described in the DO-HEALTH primary outcome paper: Bischoff-Ferrari HA et al. JAMA 2020;324(18):1855-1868.
